# Stenosis of the left pulmonary veins after atrial fibrillation catheter ablation

**DOI:** 10.31744/einstein_journal/2023AI0534

**Published:** 2023-12-12

**Authors:** Tainá Estruzani, Leonardo Kenji Nesi Mitsutake, Bruna Alexandre, Walther Yoshiharu Ishikawa, Roberto Sasdelli, Gilberto Szarf, Murilo Marques Almeida Silva, Marcelo Buarque de Gusmão Funari, Luiz Carlos Paul, Eduardo Kaiser Ururahy Nunes Fonseca

**Affiliations:** 1 Hospital Israelita Albert Einstein São Paulo SP Brazil Hospital Israelita Albert Einstein , São Paulo , SP , Brazil .

**Figure 1:**
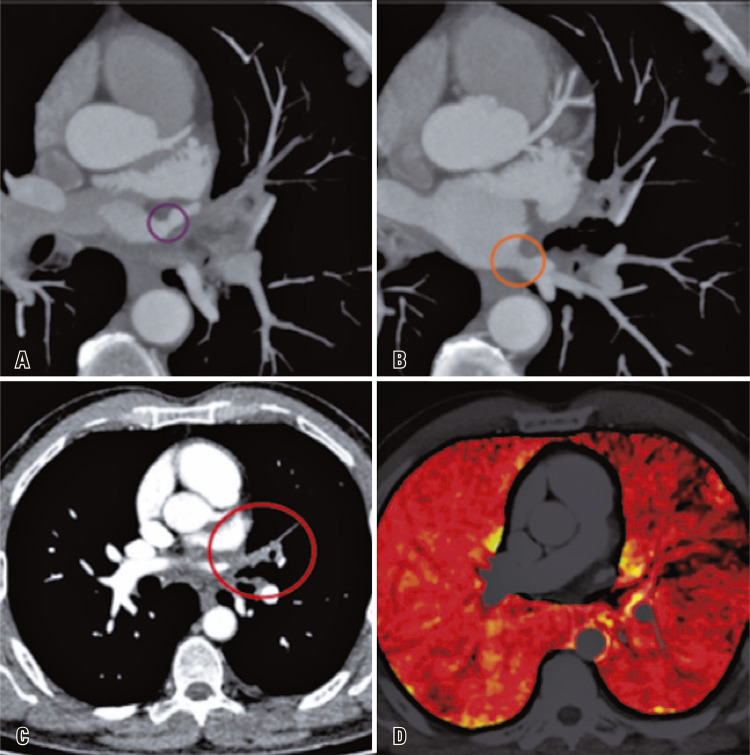
Computed tomography pulmonary angiogram demonstrates a focal reduction in the caliber of both the ostia of the left upper pulmonary vein (purple circle in image A) and left lower pulmonary vein (orange circle in image B); occlusion of pulmonary venous branches upstream of the stenotic point is observed (red circle in image C) along with corresponding segmental pulmonary hypoperfusion within the territory drained by the stenosed veins is evident in the iodine map images (image D)

A 55-year-old male patient presented to the emergency department with ventilatory-dependent chest pain, 5 months after undergoing radiofrequency ablation for atrial fibrillation. Upon initial assessment, the patient exhibited an elevated D-dimer level of 1122ng/mL. Consequently, a computed tomography pulmonary angiogram (CTPA) was requested to investigate because of the possibility of a pulmonary thromboembolism. The CTPA ( Figure 1 ) revealed a focal reduction in the caliber of both the left upper and lower pulmonary veins ostia. Additionally, occlusion of the pulmonary venous branches upstream of the stenotic point was observed. The corresponding segmental pulmonary hypoperfusion within the territory drained by the stenosed veins was clearly illustrated in the iodine map images.

**Figure 2:**
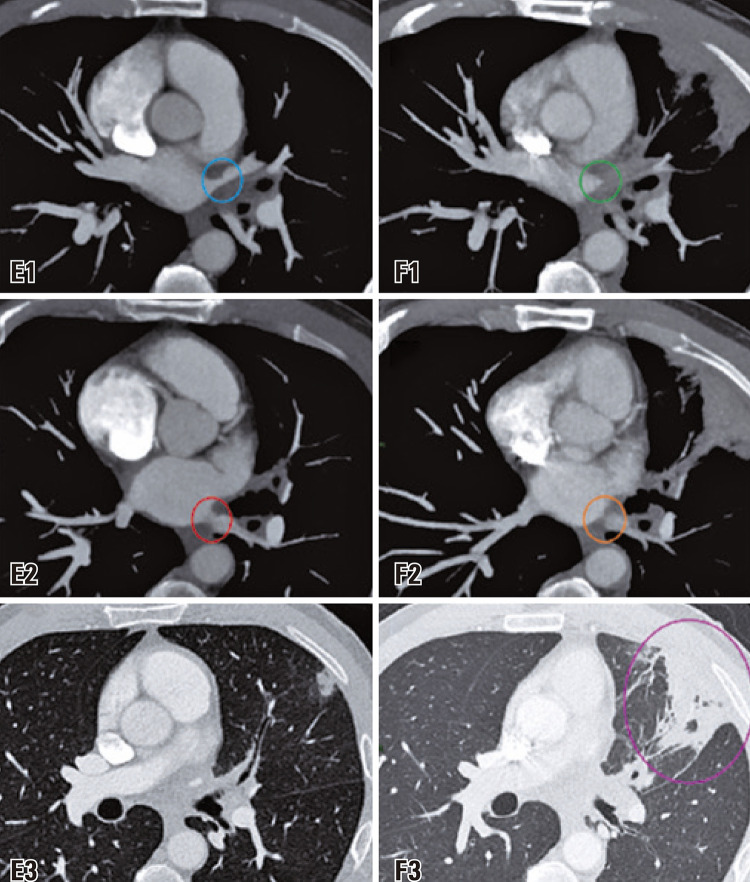
Control computed tomography pulmonary angiogram. The initial examination (images in column E) and a follow-up study after 3 months (images in column F) illustrate the progressive stenosis of the left inferior pulmonary vein (orange circle in image F2) and more extensive stenosis in the superior venous branch (green circle in image F1), which is now occluded. This occlusion is associated with the presence of a pulmonary infarction area (pink circle in image F3)

After 3 months, a follow-up CTPA ( Figure 2 ) was performed, which revealed the progression of the stenosis and the emergence of wedge consolidation in the drainage territory, indicative of an infarction area.

**Figure 3:**
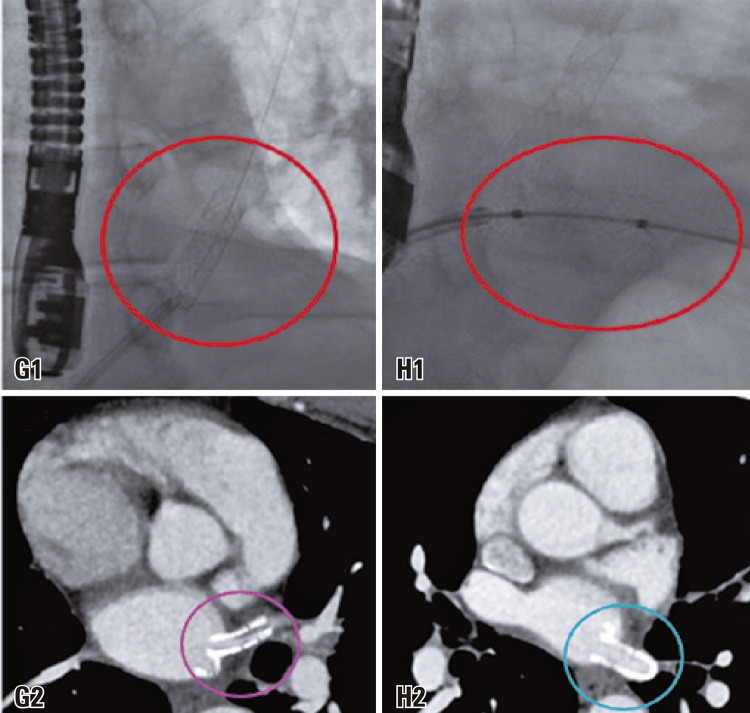
Stent placement within the upper pulmonary vein ostia (G1), and lower pulmonary vein ostia (H1). After 6 months, a subsequent computed tomography pulmonary angiogram examination reveals restenosis in the left upper pulmonary vein (pink circle in image G2) while demonstrating a patent inferior pulmonary vein with preserved caliber (blue circle in image H2)

The patient underwent a transluminal angioplasty with stent placement in the ostia of the left superior and inferior pulmonary veins. However, after 6 months, a follow-up chest CT angiography was performed, which revealed restenosis of the left superior pulmonary vein ( Figure 3 ).

Pulmonary vein stenosis is a potential complication of radiofrequency catheter ablation used in the treatment of atrial fibrillation. This condition arises due to thermal injury during the procedure, leading to the subsequent formation of fibrosis and tissue scarring. Consequently, the vessel caliber is reduced, resulting in stenosis. ^( 1 , 2 )^

Advancements in ablation techniques, such as temperature reduction, antral isolation, monitoring by intracardiac echocardiography, and ablation of atrial fibers in the interatrial septum and roof of the left atrium, have contributed to a reduction in the occurrence of pulmonary vein stenosis. However, its occurrence still varies incidence, ranginges from 0.4 to 0.7%. ^( 1 - 3 )^

Symptoms associated with pulmonary vein stenosis are nonspecific and include dyspnea, cough, fatigue, chest pain, and hemoptysis. To increase the detection rate, it is advisable to maintain a low threshold for evaluation using imaging techniques like CTPA. ^( 4 )^

Angioplasty followed by stent placement is the most commonly used and effective treatment option for patients with severe pulmonary vein stenosis. However, owing to the high incidence of restenosis within the stent, it is essential to maintain vigilant monitoring of patients for recurrent symptoms and, if considered essential, imaging studies should be performed promptly. ^( 1 , 5 )^

Hence, it is crucial to assess, symptomatic patients who have undergone radiofrequency ablation for pulmonary vein stenosis in order to achieve early diagnosis and prevent severe complications, including pulmonary infarction, veno-occlusive disease, and pulmonary arterial hypertension. ^( 1 )^
